# Data scheme and data format for transferable force fields for molecular simulation

**DOI:** 10.1038/s41597-023-02369-8

**Published:** 2023-07-27

**Authors:** Gajanan Kanagalingam, Sebastian Schmitt, Florian Fleckenstein, Simon Stephan

**Affiliations:** Laboratory of Engineering Thermodynamics (LTD), RPTU Kaiserslautern, Kaiserslautern, 67663 Germany

**Keywords:** Research data, Cheminformatics, Atomistic models, Computational models

## Abstract

A generalized data scheme for transferable classical force fields used in molecular simulations, i.e. molecular dynamics and Monte Carlo simulation, is presented. The data scheme is implemented in an SQL-based data format. The data scheme and data format is machine readable, re-usable, and interoperable. A transferable force field is a chemical construction plan specifying intermolecular and intramolecular interactions between different types of atoms or different chemical groups and can be used for building a model for a given component. The data scheme proposed in this work (named TUK-FFDat) formalizes digitally these chemical construction plans, i.e. transferable force fields. It can be applied to all-atom as well as united-atom transferable force fields. The general applicability of the data scheme is demonstrated for different types of force fields (TraPPE, OPLS-AA, and Potoff). Furthermore, conversion tools for translating the data scheme between .xls spread sheet format and the SQL-based data format are provided. The data format can readily be integrated in existing workflows, simulation engines, and force field databases as well as for linking such.

## Introduction

Molecular simulation is a powerful tool for predicting macroscopic thermophysical properties as well as for the modeling of nanoscopic processes. Molecular simulation, namely molecular dynamics (MD) and Monte Carlo (MC) simulation, have become an indispensable tool in many scientific disciplines such as computational physics^1–4^, physical chemistry^5–8^, molecular biology^9–13^, and engineering^14–17^. In MD and MC simulations, matter is modeled on the atomistic level based on molecular interactions, which are described by so-called force fields. A force field is the mathematical description of the molecular interactions. The quality of molecular simulation results primarily depends on the quality of the employed force field^18–24^. Hence, an important focus has been in the past decades on the force field development and, accordingly, a large number of force fields is available today^25^. Also, the development of new force fields is still a very active field. Yet, the electronic availability, transparency, and usability of molecular force fields remains unsatisfactory^26^. Despite their importance, data science aspects (databases, data formats, interoperability, ontologies, FAIR principles^27^ etc.) of force fields are still in their infancy.

While molecular interactions can be modeled today using first principle quantum mechanics, such simulation methods are computationally too expensive for the simulation of many particle systems as required for example in molecular biology. Therefore, molecular simulations based on Newton’s mechanics and classical force fields are widely used today. In classical force fields, the molecular interactions are modeled by interaction potentials describing the potential energy as a function of the distance and orientation $U(\underline{r})$. These interaction potentials provide a relatively simple approximation of the ‘true’ molecular interactions. Yet, these force fields have proven very powerful and are successfully used across many scientific fields today.

A force field is a collection of parametric equations and corresponding parameter values describing the interaction potentials between interaction sites representing atoms or groups of atoms. Force fields are used in molecular dynamics simulations to calculate forces between interaction sites. Based on these forces, the trajectories of the interaction sites are computed. Alternatively, the potential energy is directly used in Monte Carlo simulations for evaluating the probability that a given randomly generated atomistic configuration exists.

Transferable force fields for molecular substances are a particularly powerful tool as they can be used for modeling a large number of substances. A transferable force field is a generalized chemical construction plan for substance classes, e.g. characterizing the interaction between two chlorine atoms or the angle potential in an aromatic ring. Therefore, a transferable force field itself cannot be directly used for carrying out molecular simulations. However, based on a transferable force field, component-specific force fields can be uniquely derived by a user and then employed in a simulation. Hence, the strength of transferable force fields lies in their generalized description of molecular interactions, which comes at the cost of a high abstraction level and challenges in the usability.

A large number of transferable force fields, i.e. construction plans, is available today, for example DREIDING^28^, UFF^29^, AMBER^30^, PCFF8^31^, TraPPE-UA^32–43^, OPLS-AA^44–48^, Potoff^49–52^, and CVFF^53^. They are mostly used for modeling fluid states. The coverage of the transferable force fields for modeling different types of substances strongly varies, i.e. the variety of chemical groups and interactions captured in the construction plan. For example, some force fields are restricted to hydro- or halocarbons^49^ and others cover a large range of the periodic system^44^. Hence, transferable force fields can consist of hundreds of parameters. Moreover, these parameter data are heterogeneous as the potentials of a transferable force field describe different types of interactions, e.g. intermolecular and intramolecular.

Different data aspects of molecular simulations have been addressed in recent years for increasing the transparency, reproducibility^26,54–56^, and interoperability of molecular simulations^57–65^. Yet, these attempts mostly focus on the simulation scenario setup and the simulation results. Thereby, multiple data formats for atomistic configurations, i.e. snapshots of simulations, have been well established, e.g. the *.xyz* file format or the *.pdb* file format for proteins^66^. Also, data formats for specific individual molecules are available which includes data formats for (small) molecules such as CML^67^ format, SYBYL Line Notation^68^, SMIRNOFF format^69^, MCDL^70^, and SMILES^71^ as well as for macromolecules such as proteins, peptides, and polymers such as HELM^72^ and SPICES^73^. Moreover, some transferable force fields are electronically accessible for users, e.g. the CHARMM force field in ref. ^74^, the Amber force field in ref. ^75^, the AMOEBA force field in ref. ^75^, the TraPPE force field in refs. ^76,77^, the Merck force field in ref. ^78^, and the OPLS force field in refs. ^77,79^. Yet, most of these use individual data formats designed for the respective force field or computational framework. Also, most of these tools provide component-specific force field files (built from an implemented transferable force field), i.e. they are atom typing tools for generating force fields for a given individual molecule. The OpenKIM^80^, the OpenMM^75,81^, and the MoSDeF^59,77,82^ platform provide a digital infrastructure for atom typing and storing force field parameters, which can also be used for different molecular modeling and simulations tasks, e.g. setting up simulation scenarios and coupling with simulation engines.

For building a component-specific force field from a transferable force field construction plan, multiple challenges arise. Publications on transferable force fields use many different notations, units systems, mathematical forms of interaction potentials etc., which makes it difficult to use different force fields in one workflow. Also, the atomistic coordinates of the interaction sites in a molecule are only implicitly described by transferable force fields by the global minimum of the intramolecular interaction potentials. Moreover, different atomistic configurations, i.e. conformations, of a given molecule are often feasible and the equilibrium conformation (or distribution of conformations) is usually not a priori known. Furthermore, several force field features are treated and implemented differently in different simulation engines, e.g. electrostatic multipoles, long-range forces, and rigidity constraints, which can cause deviations in the results^54^. Moreover, important differences are present in the design concepts of different transferable force fields, which makes switching from one to another transferable force field in a workflow tedious and error-prone. Accordingly, there are only very few force field databases^76,79,83^ available today, which mostly cover the force fields developed by the creators of the database.

**Fig. 1:**
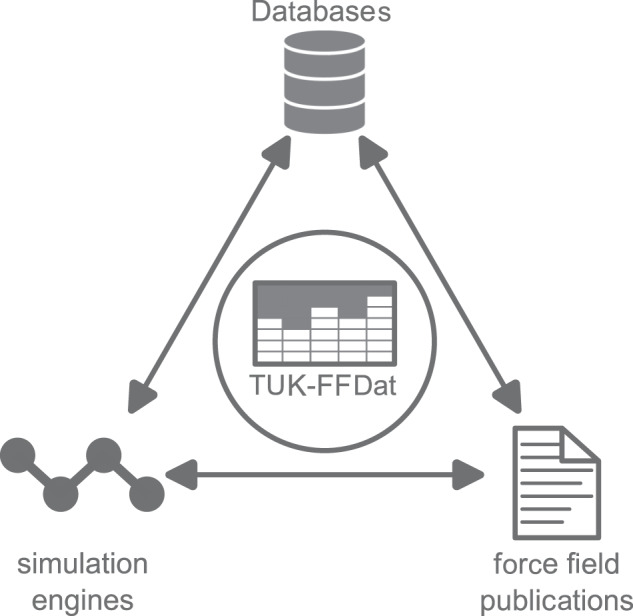
Applicability of the TUK-FFDat data scheme and data format for establishing a link between databases, simulation engines, and force field publications.

In this work, a generalized data scheme for transferable force fields is proposed, which formalizes the underlying general chemical construction plan and is applicable for a large variety of transferable force fields. Based on the developed data scheme, a concrete SQL-based data format is proposed. The data scheme developed in this work is based on identifiers that are both human-readable as well as machine-readable. The latter in particular enables the integration in automated workflows. Also, the syntax is chemically consistent such that for example bond order rules are correctly captured. The data scheme is moreover designed to be simple, flexible, and extendable. The applicability of the data scheme and data format is demonstrated for different types of transferable force fields. The data scheme and data format proposed in this work (termed TUK-FFDat) enables an interoperable data exchange between publications of new transferable force fields, users of different molecular simulation engines, and force field databases (cf. Figure 1).

This paper is organized as follows: First, different classification approaches and features of transferable force fields are introduced. Based on this ontology, the novel data scheme is built. Then, the implementation of the data scheme in an SQL-based data format is presented followed by an exemplary application of the presented data format to three transferable force fields. Conversion tools that translate the data scheme information from a user-friendly .xls spread sheet format to the SQL database format is described in the Methods section.

## Results

### Classification of force fields

**Fig. 2:**
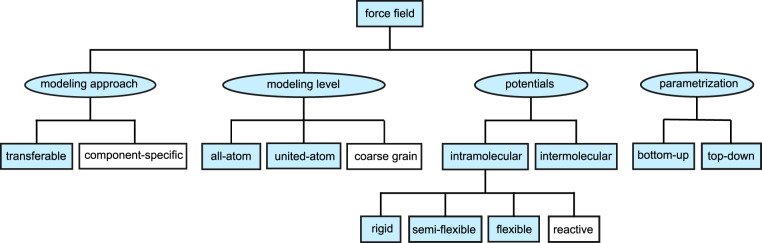
Force field ontology and classification used in this work. Blue indicates attributes covered by the TUK-FFDat data scheme and data format.

Force fields can be classified using different attributes. Figure 2 shows a systematic classification of force fields regarding the modeling approach, the model detail level, the interaction potential types, and the parametrization approach. Blue highlights in the ontology (Figure 2) indicate the coverage of the data scheme developed in this work.

There are two main modeling approaches for molecular force fields: (i) component-specific, where the layout of the interaction sites, the choices for the parameter functions as well as the parametrization procedure is carried out for a specific substance, e.g. ethanol. This usually results in a relatively accurate model since the focus was on that substance alone. The downside of that approach is that the developed model is only valid for that substance and no parts of the model can in general be transferred and re-used for modeling other substances. In the transferable force field approach (ii), molecular features and interactions are modeled in a generalized way based on building blocks, e.g. single atoms or groups of atoms. These force fields will usually (but not necessarily) be less accurate than component-specific force fields for a given substance since the objective during the development was broader. Yet, transferable force fields can be applied in a wider sense since the molecular features are captured in building blocks.

**Fig. 3:**
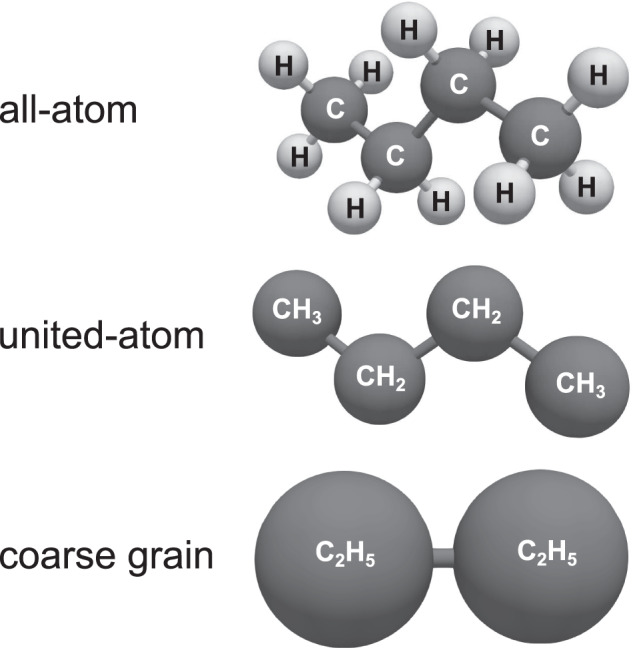
Classification of force fields according to the modeling level used to model molecules based on interaction sites (spheres).

Different modeling levels can be used for developing force fields, namely (i) all-atom; (ii) united-atom; and (iii) coarse grain. Figure 3 shows these different approaches – using *n*-butane as an example. Going from (i) to (iii), the degree of abstraction of the molecular model increases, which also increases the computational efficiency as less details are included. However, the accuracy for predicting macroscopic thermophysical properties does not necessarily depend on the degree of abstraction^19,84^. Usually, the ability to extrapolate to state regions that were not considered in the fit usually decreases with increasing the degree of abstraction. In all-atom force fields, each atom in a molecule is explicitly modeled by an interaction site, including small hydrogen atoms. In united-atom force fields, small groups of atoms are modeled as an interaction site. In this approach, usually, chemical groups, e.g. methyl or methylene groups, are fused to a single interaction site, cf. Figure 3. In united-atom force fields, especially hydrogen atoms are often substituted within the nearest larger neighbor atom. In coarse grain force fields, larger sections of molecules (or even multiple molecules) are modeled as an interaction site, cf. Figure 3. For each modeling level, an interaction site is represented by a geometrical point. However, in visualizations, interaction sites are usually represented by spheres, cf. Figure 3, representing the extend of the repulsive interactions of the respective potential (in a simplified way).

The mathematical form of the interaction potentials is an important force field attribute (cf. Figure 2). Interaction potentials are parametric functions that describe the potential energy between the interaction sites. Both intramolecular interaction potentials (between sites of the same molecule) and intermolecular interaction potentials (between sites of different molecules) exist, cf. Figure 2. The intramolecular interaction potentials establish the molecule flexibility and allow molecular vibrations. Different types of intramolecular interactions can be applied for a force field: A molecule can be fully *flexible*, meaning that all interaction sites have three independent translational degrees of freedom. Force fields that have intramolecular potentials, but have certain fixed bond lengths, fixed bond angles, or fixed torsion angles are called *semi-flexible*. Thereby, stretching between direct neighbor interaction sites is often constraint to be rigid (this allows the use of a larger time step and faster exploration of the phase space^25^). In the limiting case where all intramolecular interactions are constraint, the force field is *rigid* and no intramolecular degrees of freedom, i.e. no change in the molecular geometry and vibrations, occur. This is usually only meaningful for relatively small molecules. *Reactive* force fields are a special type of flexible force fields. In reactive force fields^85^, bonds are modeled by bond order potentials, which describe the state of a bond between two interaction sites. This enables a dynamic mapping of interaction sites during a simulation and thereby chemical reactions. Most available transferable force fields are of the flexible or semi-flexible type.

**Fig. 4:**
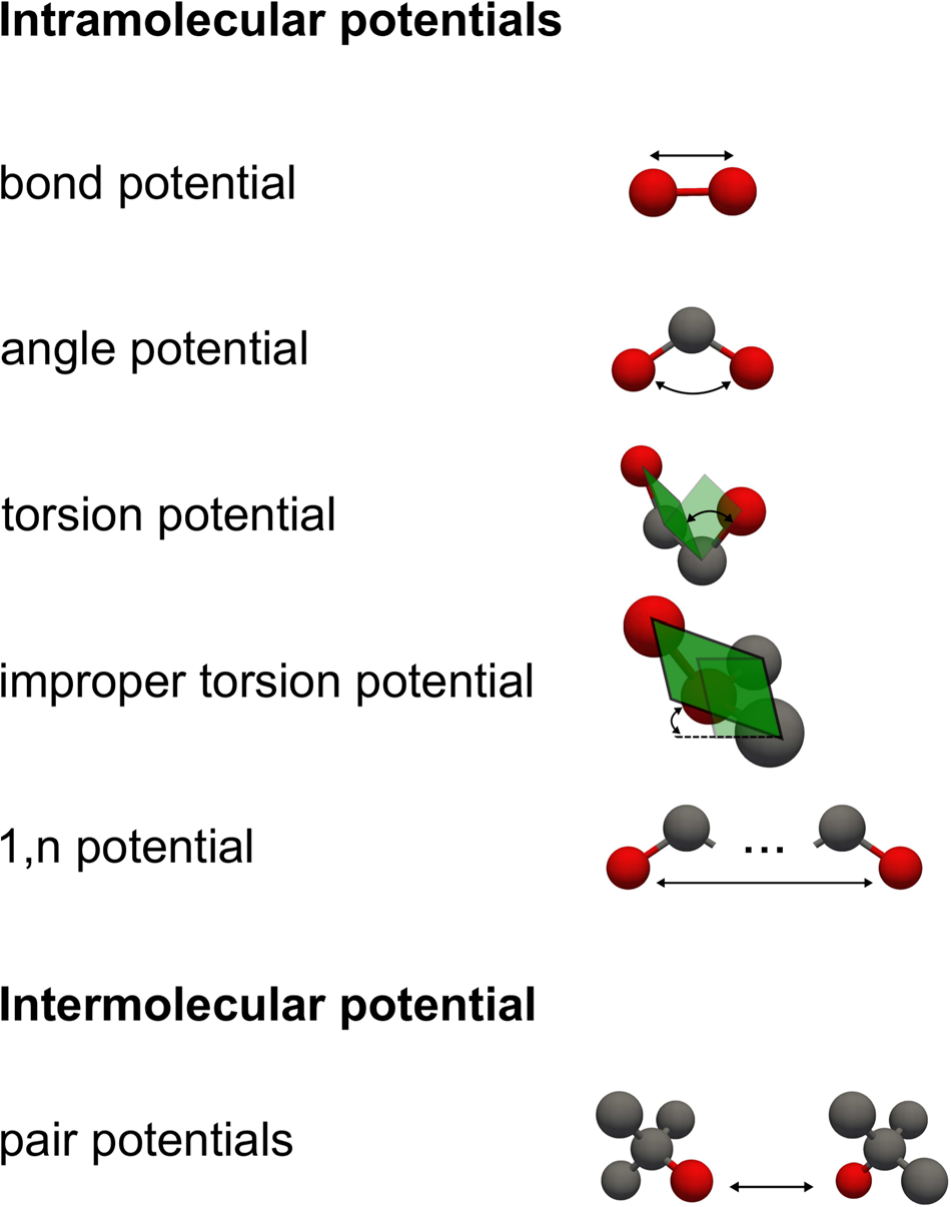
Classification of force fields based on the potential types.

Force fields consist of different types of intramolecular and intermolecular interaction potentials, Figure 4. For fully flexible force fields, different types of intramolecular potentials can occur: Interaction potentials describing the potential energy between two bonded interaction sites are called *bond potentials *– modeling a strongly localized chemical bond^86^. Bond potentials are parametric functions that usually depend on the bond length of the bond between the interaction sites under consideration. Intramolecular potentials describing the potential energy between three directly neighbored interaction sites are called *angle potentials*. The angle potentials are a function of the angle between three sites. Intramolecular potentials describing the potential energy between four directly neighbored interaction sites (for example the four carbon atoms in *n*-butane, cf. Figure 3) are called *torsion potentials*. Dihedral potentials have an important impact on the molecular configurations and the macroscopic thermophysical properties. In force fields describing branched molecules, so-called *improper torsion potentials* are used at times. These potentials describe the potential energy between four directly neighbored interaction sites, whereby three interaction sites are bonded to a fourth central interaction site. Improper torsion or dihedral potentials are usually formulated as a function of the ‘out of plane’ angle, cf. Figure 4. Intramolecular potentials describing the potential energy between two interaction sites that belong to the same molecule and have a distance of *n*−1 bonds, are called 1, *n* interaction potentials (where *n* > 1). The 1, *n* potentials model dispersive and repulsive interactions between interaction sites in a molecule that are not close neighbors. This is particularly relevant for large curled molecules. Usually, the 1, *n* interactions are described by scaled intermolecular potentials (see below). The van der Waals and the electrostatic interactions are usually scaled individually.

There are (in practically all cases) two types of intermolecular interactions: Electrostatic interactions, dispersive (attractive) interactions, and repulsive interactions. The latter two model attractive forces at moderate distances (a.k.a. van der Waals forces) and repulsive forces at short distances (mimicking the overlap of electron orbitals)^25,86^. In most cases, effective pair potentials are used for describing intermolecular interactions. For these interactions, mostly the Lennard-Jones^87–89^ potential or the Mie^90^ potential is used. The electrostatic interactions are mostly modeled by simple point charges, but also higher multipole interaction sites are used in force fields at times. These relatively simple electrostatic interactions model the molecular orbital charge distribution (that is in reality much more complex), e.g. the charge distribution in alcohol groups and *π*-orbitals in aromatic components. To describe the potential energy between different types of interaction sites (kinds of atoms or groups of atoms), in practically all cases, the same mathematical functions are used within a given transferable force field and the cross-interaction parameters are determined using combination rules.

Both the intermolecular and the intramolecular potential functions have parameters that – together – describe the chemical and physical nature of the interactions. For the development of force fields, different strategies for determining the parameter values have been applied in the literature (cf. Figure 2). Two main routes are established today: (i) a bottom-up approach and (ii) a top-down approach.

In the bottom-up approach, the ‘true’ molecular interactions are determined using quantum mechanical simulations^91–94^. Based on the results, both the intermolecular and the intramolecular interactions in force fields can in general be determined. The parameter values of the intramolecular potentials are often fitted to first principle quantum chemical simulation results for the potential energy surface (PES). Yet, using quantum mechanical simulations for fitting the intermolecular potential parameters is conceptually and computationally challenging, e.g. since multi-body interactions are mapped to pair interactions.

In the top-down approach, the parameter values of the potential functions are determined using macroscopic thermophysical property data. The parameters are tuned such that the force field describes a given set of macroscopic properties well. For force fields for fluids, mostly vapor–liquid equilibrium properties and self-diffusion data is used for the parametrization. In many cases, the top-down approach and the bottom-up approach are combined such that intramolecular interactions are determined from quantum chemical simulation results and intermolecular interactions using macroscopic thermophysical property data.

Furthermore, force fields can be sub-classified based on the mathematical functions employed in a force field. Also, machine learning force fields have been developed in recent years as a novel class^95^. In machine learning force fields, the potential functions and their parameters are determined using machine learning (mostly using large PES data sets). Machine learning force fields can be considered a sub-type of the bottom-up parametrization strategy.

The generalized data scheme proposed in this work captures a large variety of transferable force field types (blue highlighting in Figure 2). Based on the ontology and terminology introduced in Figure 2, the new data scheme is presented in the following.

### Definition of data scheme

**Fig. 5:**
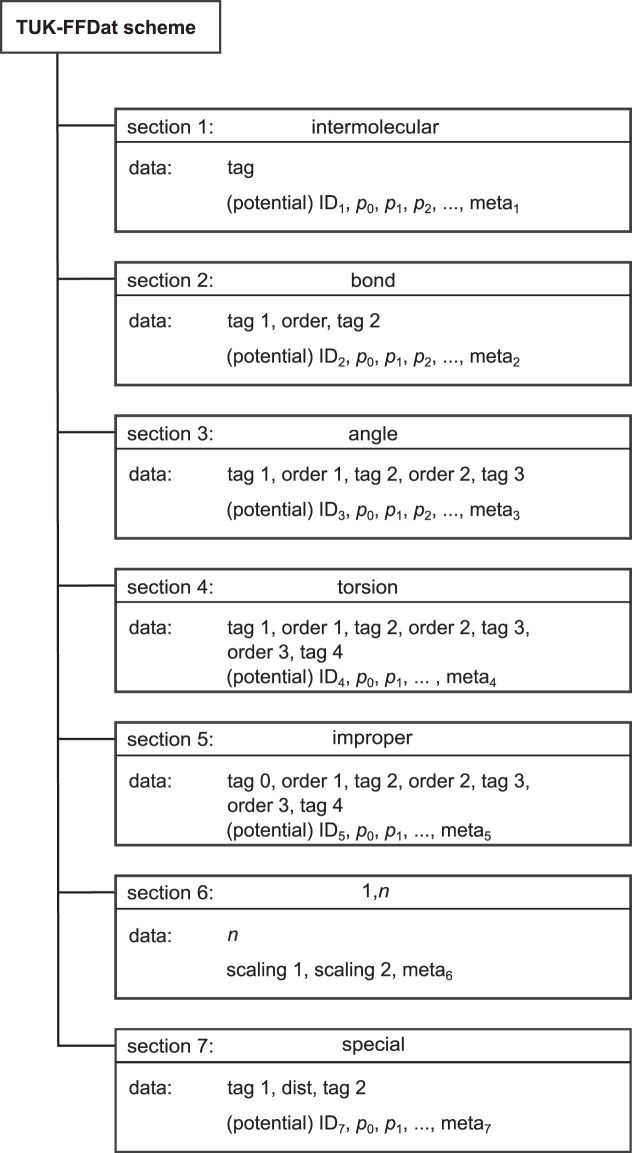
Schematic overview of TUK-FFDat data scheme for transferable force fields.

The data scheme proposed in this work consists of seven sections that formalize the definition of a transferable force field construction plan. Figure 5 gives an overview of the data scheme. In the *i* = 1‥.7 sections, the interaction potentials constituting a transferable force field are stored as follows: (1) intermolecular interactions; (2) bond intramolecular interactions; (3) angle intramolecular interactions; (4) torsion intramolecular interactions; (5) improper intramolecular interactions; (6) 1, *n* interactions; and (7) special case interactions.

A ‘tag’ notation is introduced defining the interaction site type, i.e. atom or group of atoms (in the case of a united-atom force field). Tag tuples are used in the different sections to indicate the combination of interaction site types defining a specific interaction, e.g. a bond between a hydrogen atom and a carbon atom. Using the tag notation and the bond order between the interaction sites, the interaction potentials acting between a given set of sites is defined in a generalized way.

**Fig. 6:**
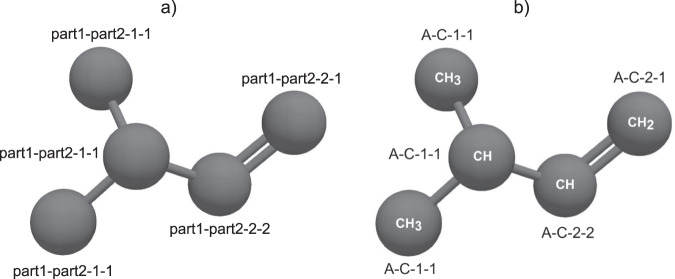
Exemplaric definition of tag identifier notation (cf. Table 1) for interaction sites (atoms or groups of atoms) using 3-methyl-1-butene: (**a**) last two parts of the tag specifying bond structure in a molecule (details given in the text); (**b**) first two parts of the tag specifying the atom type and site structure of the model.

A tag consists of four parts that are separated by a hyphen ‘-’. The first two parts are strings and the third and fourth part are integer values. Details are given in Table 1. Figure 6 shows a united-atom 3-methyl-1-butene (C_5_H_10_) molecule model illustrating the definition of the tag. The first part of the tag is an abbreviation representing the functional group to which the interaction site is assigned. Table 2 gives a list of chemical groups and their abbreviations used in the data scheme. The second part of the tag indicates the type of atom or group of atoms modeled by the interaction site under consideration. For atoms, the classical periodic table notation is used^96^. For sites modeling a group of atoms (in an united-atom force field), fused hydrogen and carbon atoms are indicated by a ‘C’. Hence, in this part of the tag hydrogen atoms are neglected in united-atom models unless a site explicitly models a single hydrogen atom. The third part of the tag is the number of bonds the interaction site forms with other (non-hydrogen) interaction sites. The fourth part of the tag indicates the highest bond order the interaction site under consideration enters into. The tag ‘A-C-2-1’, cf. Figure 6, for example indicates a carbon atom C (fused with the substituted hydrogen atoms) in an alkane group A forming one ‘1’ bond with (non-hydrogen) interaction sites, which has a bond order of ‘2’, i.e. a double bond. The tag notation also enables a direct distinction of a particular atom type that is modeled differently, i.e. different parameters, in different chemical environments. Details on the tag notation are given in the Supplementary Material.

**Table 1:** Definition of tag notation part1-part2-part3-part4 characterizing a given interaction site and data type of the individual tag entries.

part	value	description
part1	string	functional group of which interaction site is part of (cf. Table 2)
part2	string	atom or group of atoms modeled by interaction site
part3	integer	number of bonds of interaction site (with non-hydrogen atoms)
part4	integer	highest bond order of interaction site

**Table 2:** Functional groups included in the data scheme (first part of the tag, cf. Table 1).

abbreviation	type	functional group
A*	CH_*x*_–CH_*x*_^*a*^, CH_*x*_ = CH_*x*_^*b*^, CH_*x*_ ≡ CH_*x*_^*c*^	alkane
Ac	CH_*x*_–O–C(=O)–CH=CH_2_^*a*^	acrylate
Ace	CH_*x*_–O–C(–*X*)_2_–O—CH_*x*_^*a,b*^	acetal
Ad	CH_*x*_–C( = O)–N–*X*_2_^*a, d*^	amide
Ak	CH_*x*_–O–H^*a*^	alcohol
Al	*X*–C(–H) = O^*a, b*^	aldehyde
Am	CH_*x*_–N–*X*_2_^*a, d*^	amine
B**	CH–CH (arom.)	benzene
CA**	CH_2_—CH_2_ (cyc.)	cycloalkane with 6 < (ring size) < 18
CA5**	CH_2_—CH_2_ (cyc.)	cycloalkane with ring size 5
CA6**	CH_2_—CH_2_ (cyc.)	cycloalkane with ring size 6
Cac	CH_*x*_–C( = O)–O—H^*a*^	carboxylic acid
DS	CH_*x*_–S–S–CH_*x*_^*a*^	disulfide
E	CH_*x*_–O–CH_*x*_^*a*^	ether
Es	CH_*x*_–C( = O)–O–CH_*x*_^*a*^	ester
K	CH_*x*_–C( = O)–CH_*x*_^*a*^	ketone
mAc	CH_*x*_–O–C( = O)–C(–CH_3_) = CH_*x*_^*a*^	methacrylate
Nl	CH_*x*_–C ≡ N^*a*^	nitrile
No	CH_*x*_–N–O_2_^*a*^	nitro
Sd	CH_*x*_–S–CH_*x*_^*a*^	sulfide
Tl	CH_*x*_–S—H^*a*^	thiol

^*a*^*x* ∈ [0, 1, 2, 3], ^*b*^*x* ∈ [0, 1, 2], ^*c*^*x* ∈ [0, 1], ^*d*^*X* ∈ [H, CH_*x*_].

*Both, alkenes (sp^2^) and alkynes (sp^1^) are abbreviated ‘A’ in the first part of the tag.

**Functional groups inside cycloalkanes or aromatic benzene rings are also abbreviated

‘CA’ and ‘B’, respectively, in the first part of the tag.

In the seven sections of the data scheme (cf. Figure 5), chemical sub-structures (i.e. formations of two sites (bonds), three sites (angles) etc.) are characterized using tuples of tags indicating the participating interaction sites. This constitutes the chemical construction plan. Each of the seven sections of the data scheme has a list of entries defining the interaction potentials and their parameters assigned to a given chemical structure, i.e. combination of types of interaction sites. The interaction potentials are represented by parametric functions with the parameters *p*_0_, *p*_1_,…, *p*_*n*_ (cf. Figure 5). The mathematical functions used for describing a given interaction are represented by the ‘ID_*i*_’ with *i* = 1.‥7. Each section has its own ID and interaction potential list. For example, for the bond potential *i* = 2, the classical harmonic function has the ID_2_ = 1. Moreover, meta data indicating the origin of the data (in most cases the parameter values) is appended for each structural information. For this purpose, the DOI numbers are used as references, which provide a unique link to the respective references^97^.

In the following, the structure and syntax of each of the seven sections is introduced in detail. It should be noted that the equilibrium structure (bonds, bond angles,…) of a given molecule is implicitly given by a global minimum of its total potential energy, which is therefore not explicitly described by the data scheme.

The first section of the data scheme is termed *intermolecular* and contains the information on the intermolecular interaction potentials between interaction sites. The assignment of the individual intermolecular potential functions by the corresponding IDs is given in Table 3. The *intermolecular* section explicitly lists potential functions with its corresponding parameters and a combination rule. The interaction sites in the first section of the data scheme are defined by a single corresponding tag. The potential functions used for modeling the interactions between given site types are encoded in the ID_1_ (cf. Table 3). Also the combination rule type describing the interaction potential between unlike interaction sites is comprised in the ID_1_. For a given transferable force field, the ID_1_ is constant. In the list of intermolecular interaction potential functions (cf. Table 3), also the meaning of the parameter values is specified.

**Table 3:** Intermolecular potential functions and their parameters (first section of data scheme, cf. Figure 5), where *r*_*ij*_ indicates the distance between the considered interaction sites *i* and *j*, *ε*_0_ the electric constant, *k*_B_ the Boltzmann constant, *q* the charge, *ε* the dispersion energy, *σ* the size parameter, and *n* the potential exponent.

ID_1_	function	p_1_	p_2_	p_3_	p_4_
1	$4{\varepsilon }_{ij}\left[{\left(\frac{{\sigma }_{ij}}{{r}_{ij}}\right)}^{12}-{\left(\frac{{\sigma }_{ij}}{{r}_{ij}}\right)}^{6}\right]+\frac{1}{4{\varepsilon }_{0}\pi }\frac{{q}_{ij}}{{r}_{ij}}$	*q*_*ii*_	*ε*_*ii*_	*σ*_*ii*_	—
	with:				
	$${q}_{ij}={q}_{ii}{q}_{jj},{\varepsilon }_{ij}=\sqrt{{\varepsilon }_{ii}{\varepsilon }_{jj}},{\sigma }_{ij}=\frac{{\sigma }_{ii}+{\sigma }_{jj}}{2}$$				
2	${C}_{n}{\varepsilon }_{ij}\left[{\left(\frac{{\sigma }_{ij}}{{r}_{ij}}\right)}^{{n}_{ij}}-{\left(\frac{{\sigma }_{ij}}{{r}_{ij}}\right)}^{6}\right]+\frac{1}{4{\varepsilon }_{0}\pi }\frac{{q}_{ij}}{{r}_{ij}}$	*q*_*ii*_	*ε*_*ii*_	*σ*_*ii*_	*n*_*ii*_
	with:				
	${n}_{ij}=\frac{{n}_{ii}+{n}_{jj}}{2}$, ${C}_{n}=\left(\frac{{n}_{ij}}{{n}_{ij}-6}\right){\left(\frac{{n}_{ij}}{6}\right)}^{\frac{6}{{n}_{ij}-6}}{q}_{ij}={q}_{ii}{q}_{jj}$, ${\varepsilon }_{ij}=\sqrt{{\varepsilon }_{ii}{\varepsilon }_{jj}},{\sigma }_{ij}=\frac{{\sigma }_{ii}+{\sigma }_{jj}}{2}$				
3	$4{\varepsilon }_{ij}\left[{\left(\frac{{\sigma }_{ij}}{{r}_{ij}}\right)}^{12}-{\left(\frac{{\sigma }_{ij}}{{r}_{ij}}\right)}^{6}\right]+{e}^{2}\frac{{q}_{ij}}{{r}_{ij}}$	*q*_*ii*_	*ε*_*ii*_	*σ*_*ii*_	—
	with:				
	$${q}_{ij}={q}_{ii}{q}_{jj},{\varepsilon }_{ij}=\sqrt{{\varepsilon }_{ii}{\varepsilon }_{jj}},{\sigma }_{ij}=\frac{{\sigma }_{ii}+{\sigma }_{jj}}{2}$$				
4	${\varepsilon }_{ij}\left[{\left(\frac{{r}_{{\rm{\min }},ij}}{{r}_{ij}}\right)}^{12}-{\left(\frac{{r}_{{\rm{\min }},ij}}{{r}_{ij}}\right)}^{6}\right]+\frac{1}{{\varepsilon }_{l}}\frac{{q}_{ij}}{{r}_{ij}}$	*q*_*ii*_	*ε*_*ii*_	*r*_min, *ii*_	—
	with:				
	$${q}_{ij}={q}_{ii}{q}_{jj},{\varepsilon }_{ij}=\sqrt{{\varepsilon }_{ii}{\varepsilon }_{jj}},{r}_{{\rm{\min }},ij}=\frac{{r}_{{\rm{\min }},ii}+{r}_{{\rm{\min }},jj}}{2}$$				

The second section of the data scheme is termed *bond* and contains the specifications for the bond potentials for different combinations of two directly neighbored interaction sites. Hence, all information on intramolecular bond potentials within the given transferable force field are stored in the second data scheme section. A bond interaction is specified by the tags of the two involved interaction sites ‘tag 1’ and ‘tag 2’ as well as the bond ‘order’ between the considered interaction sites (cf. Figure 5). The bond potential specification for two interaction sites consists of a bond potential function and its parameters – analogously to the intermolecular potential section. The bond potential function is encoded by the ID_2_. Details on the potential functions are given in Table 4.

**Table 4:** Bond potential functions and their parameters (second section of data scheme, cf. Figure 5), where *r*_*ij*_ is the distance between the considered interaction sites *i* and *j*, and *k* parameters of the potentials.

ID_2_	function	p_1_	p_2_	p_3_	p_4_
1	$\frac{{k}_{2}}{2}{\left({r}_{ij}-{r}_{0}\right)}^{2}$	*k*_2_	*r*_0_	—	—
2	${k}_{2}{\left({r}_{ij}-{r}_{0}\right)}^{2}+{k}_{3}{\left({r}_{ij}-{r}_{0}\right)}^{3}+{k}_{4}{\left({r}_{ij}-{r}_{0}\right)}^{4}$	*k*_2_	*k*_3_	*k*_4_	*r*_0_
3	$\frac{{k}_{4}}{4}{\left({r}_{ij}^{2}-{r}_{0}^{2}\right)}^{2}$	*k*_4_	*r*_0_	—	—

The third section of the data scheme is termed *angle*. It contains the specifications for the angle potentials for different combinations of three directly neighbored interactions sites. An angle interaction potential is specified by the tags of the three involved types of interaction sites ‘tag 1’, ‘tag 2’, and ‘tag 3’ and the two bond orders ‘order 1’ and ‘order 2’. The ‘order 1’ indicates the bond order between the central interaction site indicated by ‘tag 2’ and the first interaction site ‘tag 1’. The ‘order 2’ indicates the bond order between the ‘tag 2’ and ‘tag 3’ interaction sites. The interaction potential functions are encoded by the ID_3_. The list of mathematical functions and the corresponding parameters is given in Table 5.

**Table 5:** Angle potential functions and their parameters (third section of data scheme, cf. Figure 5), where *i* and *k* are the interaction sites that are bond to the interaction site *j*, such that *i*, *j* and *k* form the bond angle Θ, *r*_*ij*_ is the distance between the interaction sites *i* and *j*, *r*_*jk*_ is the distance between the interaction sites *j* and *k*.

ID_3_	function	p_1_	p_2_	p_3_	p_4_	p_5_	p_6_	p_7_	p_8_	p_9_
1	$\frac{{l}_{2}}{2}{\left(\Theta {-\Theta }_{0}\right)}^{2}$	*l*_2_	Θ_0_	—	—	—	—	—	—	—
2	$$\begin{array}{l}{l}_{2}{\left(\Theta {-\Theta }_{0}\right)}^{2}+{l}_{3}{\left(\Theta {-\Theta }_{0}\right)}^{3}+{l}_{4}{\left(\Theta {-\Theta }_{0}\right)}^{4}+\\ {k}_{2}\left({r}_{ij}-{r}_{1}\right)\left({r}_{jk}-{r}_{2}\right)+{N}_{1}\left({r}_{ij}-{r}_{1}\right)\left(\Theta {-\Theta }_{0}\right)+\\ {N}_{2}\left({r}_{jk}-{r}_{2}\right)\left(\Theta {-\Theta }_{0}\right)\end{array}$$	*l*_2_	*l*_3_	*l*_4_	Θ_0_	*k*_2_	*r*_1_	*r*_2_	*N*_1_	*N*_2_
3	$c\frac{{\left(cos\Theta -co{s\Theta }_{0}\right)}^{2}}{2}$	Θ_0_	*c*	—	—	—	—	—	—	—

The fourth section of the data scheme is termed *torsion* and contains the specifications for the torsion potentials for different combinations of four directly neighbored in-line (no branching) interaction sites. This type of interaction is also often named dihedral. A torsion potential is specified by the tags of the four involved types of interaction sites ‘tag 1’, ‘tag 2’, ‘tag 3’, and ‘tag 4’ and the three bond orders ‘order 1’, ‘order 2’, and ‘order 3’. The interaction sites indicated by ‘tag 1’ and ‘tag 4’ are the tail interaction sites of a torsion structure; the interaction sites indicated by ‘tag 2’ and ‘tag 3’ are the central interaction sites. Accordingly, the ‘order 1’ and ‘order 3’ specify the bond order of the tail bonds of a torsion structure; the ‘order 2’ specifies the bond order of the central bond. The potential function types are encoded by the ID_4_. The list of mathematical functions and the corresponding parameters is given in Table 6. Details on the specifications of special cis/trans isomerism-dependent torsion potentials are given in the Supplementary Material.

**Table 6:** Torsion potential functions and their parameters (fourth section of data scheme, cf. Figure 5), where Φ is the torsion angle formed by the interaction sites under consideration and *c* and *n* are potential parameters.

ID_4_	function	p_1_	p_2_	p_3_	p_4_	p_5_	p_6_	p_7_	p_8_	p_9_	p_10_	p_11_	p_12_
1	$$\begin{array}{l}{c}_{0}+{c}_{1}\left(1+{\rm{c}}{\rm{o}}{\rm{s}}\Phi \right)+{c}_{2}\left(1-{\rm{c}}{\rm{o}}{\rm{s}}2\Phi \right)+\\ {c}_{3}\left(1+{\rm{c}}{\rm{o}}{\rm{s}}3\Phi \right)\end{array}$$	*c*_0_	*c*_1_	*c*_2_	*c*_3_	—	—	—	—	—	—	—	—
2	$c\frac{{\left(\Phi {-\Phi }_{0}\right)}^{2}}{2}$	*c*	Φ_0_	—	—	—	—	—	—	—	—	—	—
3	${\sum }_{i=0}^{6}\,{c}_{i}\cos \,i\Phi$	*c*_0_	*c*_1_	*c*_2_	*c*_3_	*c*_4_	*c*_5_	*c*_6_	—	—	—	—	—
4	${c}_{0}\left[1-cos\left(2\Phi {+\Phi }_{0}\right)\right]$	*c*_0_	Φ_0_	—	—	—	—	—	—	—	—	—	—
5	${\sum }_{i=0}^{7}\,{c}_{i}\,co{s}^{i}\Phi$	*c*_0_	*c*_1_	*c*_2_	*c*_3_	*c*_4_	*c*_5_	*c*_6_	*c*_7_	—	—	—	—
6	${\sum }_{i=1}^{4}{c}_{i}\left[1+\cos \left({n}_{i}\Phi {-\Phi }_{i}\right)\right]$	*c*_1_	*n*_1_	Φ_1_	*c*_2_	*n*_2_	Φ_2_	*c*_3_	*n*_3_	Φ_3_	*c*_4_	*n*_4_	Φ_4_

The fifth section of the data scheme is termed *improper*. It contains the specifications for improper torsion potentials of a branching intersection of four directly neighbored interaction sites. Hence, the improper torsion potential is specified by the four involved types of interaction sites ‘tag 0’, ‘tag 1’, ‘tag 2’, and ‘tag 3’ and the three bond orders ‘order 1’, ‘order 2’, and ‘order 3’ – as for the in-line torsion potential (see above). In a branched structure modeled by an improper torsion, one interaction site is the central one – indicated by the ‘tag 0’ in the data scheme. The three remaining interaction sites ‘tag 1’, ‘tag 2’, and ‘tag 3’ have a direct bond to the central one. Accordingly, ‘order 1’, ‘order 2’, and ‘order 3’ specify the bond order from the central interaction site to the respective neighboring interaction site. The three interaction sites indicated by ‘tag 0’, ‘tag 1’, and ‘tag 2’ span a specific plane (which is relevant for some improper torsion potential functions). The potential functions used for modeling the improper torsion differs in most cases from those used for modeling the in-line torsion. The improper torsion potential function types are encoded by the ID_5_. The list of mathematical functions and the corresponding parameters is given in Table 7.

**Table 7:** Improper torsion potential functions and their parameters (fifth section of data scheme, cf. Figure 5), where Ψ is the out of the plane angle formed by the interaction sites under consideration and *l* are potential parameters.

ID_5_	function	p_1_	p_2_
1	${l}_{2}\frac{{\left(\Psi {-\Psi }_{0}\right)}^{2}}{2}$	*l*_2_	Ψ

The sixth section of the data scheme is termed *1,n*. It contains the information on the 1, *n* intramolecular interaction potentials, i.e. the potential acting between an interaction site and its *n*th neighbor. For modeling these intramolecular interactions, scaled intermolecular potentials are used. The individual parts modeling the van der Waals interactions and the electrostatic interaction of the intermolecular potential are scaled individually. Hence, the mathematical functions are adopted from the first section, but scaled by a factor. The *1,n* section of the data scheme contains two values, i.e. *n* indicating the distance of two sites in a molecule and two corresponding ‘scaling’ values. The ‘scaling 1’ contains the information on the scaling for the van der Waals interactions and ‘scaling 2’ the information on the scaling for the electrostatic interactions. If not specified otherwise, the scaling factor is taken to be 0 for *n*≤4 and 1 for *n* > 4 for both the van der Waals and the electrostatic potentials within the data scheme.

The seventh section of the data scheme is termed *special* and contains special interaction potential cases that may occur in specific transferable force fields that are not covered within the sections one to six. The syntax used for the special potential cases is similar to the *1,n* interactions introduced above. Hence, special interaction potentials are specified between two interaction sites. Special potentials model the potential energy between specific interaction sites, which have a certain distance with respect to direct bonding neighbors. The information structure in the *special* potential section is similar to the *bond* section. A *special* interaction is specified by the tags of the two involved types of interaction sites ‘tag 1’, ‘tag 2’, and ‘dist’ (cf. Figure 5). The latter specifies distance of the involved sites by counting the number of direct bonds between the sites ‘tag 1’ and ‘tag 2’. The potential functions and the corresponding parameters are encoded by the ID_7_. The list of mathematical functions and the corresponding parameters is given in Table 8. The dimensions of the parameters used in Tables 3–8 are given in Table 9.

**Table 8:** Special potential functions and their parameters (seventh section of data scheme, cf. Figure 5), where *r*_*ij*_ indicates the distance between the considered interaction sites *i* and *j*, and *k* parameters of the potentials.

ID_7_	function	p_1_
1	$\frac{{k}_{12}}{{r}_{ij}^{12}}$	*k*_12_

**Table 9:** Force field parameters (cf. Tables 3–8) and their physical dimensions as well as their units used in the TUK-FFDat data format.

parameter	dimension	unit
*ε*_*ii*_, *c*	energy	eV
*σ*, *r*	length	Å
*n*	n	1
*q*	charge	e
*k*_*i*_	energy/length^*i*^	eV/Å^*i*^
*l*_*i*_	energy/angle^*i*^	eV/deg^*i*^
Θ, Φ, Ψ	angle	deg
*N*	energy/(angle length)	eV/(Å deg)

The seven data scheme sections generalize and formalize a transferable force field construction plan. Therein, for a given transferable force field, the ID-vector **ID** = {ID_1_, ID_2_… ID_7_} specifies the mathematical structure of the model. The outlined data scheme can be applied to all-atom and united-atom force fields. Also, force fields parameterized by the bottom-up and top-down approach can be described using the data scheme. Regarding the molecular architecture and potentials, rigid, flexible, and semi-flexible force fields can be described by the data scheme. For semi-flexible force fields it is possible that individual bond lengths, bond angles or torsion angles are constrained. Details are given in the Supplementary Material.

The tag notation in combination with the bond order and the systematization of the potential types provides a formalization for transferable force field construction plans. The proposed data scheme can be used for electronically documenting and defining a large variety of transferable force fields, cf. Figure 2. Therefore, the data scheme is implemented in an SQL-based data format.

### SQL-based data format

The data scheme introduced above is implemented as an SQL-based data format to make it interoperable and directly usable in automated workflows, e.g. in simulation engines, databases, and for publishing new transferable force fields.

The information contained in each of the seven sections of the data scheme is translated into an SQL table structure in the data format. The data comprised in each of the sections of the data scheme (cf. Figure 5) are translated to the columns of the tables. The tag notation (cf. Table 1) introduced above is used for specifying interaction sites within the tables.

The data format syntax and data type used in the seven tables is specified in Tables 10, 11. For each table, the name of each column and the data type (string, real number, integer, etc.) stored in the column is specified in Tables 10, 11.

**Table 10:** Data structure of TUK-FFDat data format (Part A).

column	value	description
First table: intermolecular		
tag	tag	tag of atom or group of atoms of interaction site (cf. Table 1)
ID1	integer	identifier for potential function for intermolecular interactions and combining rule encoded in ID_1_ (cf. Table 3)
p1	real number	parameter of intermolecular potential function
p2	real number	parameter of intermolecular potential function
…	…	…
ref	string	DOI of the reference in which the potential parameters were published
Second table: bond		
tag1	tag	tag of interaction site (cf. Table 1) involved in the considered bond
order	integer	bond order of considered bond
tag2	tag	tag of interaction site (cf. Table 1) involved in the considered bond
ID2	integer or “none”	identifier for bond potential function encoded in ID_2_, cf. Table 4 (“none” indicating a fixed bond length)
p1	real number	if ID2 = = ‘none’: bond length, else: parameter of bond potential function
p2	real number	parameter of bond potential function
…	…	…
ref	string	DOI of the reference in which the potential parameters were published
Third table: angle		
tag1	tag	tag of central interaction site (cf. Table 1) involved in the considered angle
order1	integer	bond order of the bond between the sites represented by tag1 and tag2
tag2	tag	tag of interaction site (cf. Table 1) involved in the considered angle
order2	integer	bond order of the bond between the sites represented by tag2 and tag3
tag3	tag	tag of the interaction site (cf. Table 1) involved in the considered angle
ID3	integer or “none”	identifier for angle potential function encoded in ID_3_, cf. Table 5 (“none” indicating a fixed bond angle)
p1	real number	if ID3 = = ‘none’: bond angle, else: parameter of angle potential function
p2	real number	parameter of angle potential function
…	…	…
ref	string	DOI of the reference in which the potential parameters were published
Fourth table: torsion		
tag1	tag	tag of interaction site (cf. Table 1) involved in the considered torsion angle
order1	integer	bond order of the bond between the sites represented by tag1 and tag2
tag2	tag	tag of interaction site (cf. Table 1) involved in the considered torsion angle
order2	integer	bond order of the bond between the sites represented by tag2 and tag3
tag3	tag	tag of interaction site (cf. Table 1) involved in the considered torsion angle
order3	integer	bond order of the bond between the sites represented by tag3 and tag4
tag4	tag	tag of interaction site (cf. Table 1) involved in the considered torsion angle
ID4	integer or “none”	identifier for torsion angle potential function encoded in ID_4_, cf. Table 6 (“none” indicating a fixed torsion angle)
p1	real number	if ID4 = = ‘none’: torsion angle, else: parameter of torsion potential function
p2	real number	parameter of torsion potential function
…	…	…
ref	string	DOI of the reference in which the potential parameters were published

**Table 11:** Data structure of TUK-FFDat data format (Part B).

column	value	description
Fifth table: improper		
tag0	tag	tag of central interaction site (cf. Table 1) involved in the considered improper torsion angle
order1	integer	bond order of the bond between the sites represented by tag0 and tag1
tag1	tag	tag of interaction site (cf. Table 1) involved in the considered improper torsion angle
order2	integer	bond order of the bond between the sites represented by tag0 and tag2
tag2	tag	tag of interaction site (cf. Table 1) involved in the considered improper torsion angle
order3	integer	bond order of the bond between the sites represented by tag0 and tag3
tag3	tag	tag of interaction site (cf. Table 1) involved in the considered improper torsion angle
ID5	integer or “none”	identifier for improper torsion angle potential function encoded in ID_5_, cf. Table 7 (“none” indicating a fixed improper torsion angle)
p1	real number	if ID5 = = ‘none’: improper torsion angle, or: parameter of improper torsion potential function
p2	real number	parameter of improper torsion potential function
…	…	…
ref	string	DOI of the reference in which the potential parameters were published
Sixth table: 1n_potential		
n	integer	distance between the two sites involved in the 1, *n* potential given in number of bonds between them
scaling1	real number	scaling factor applied to the potential modeling van der Waals interactions
scaling2	real number	scaling factor applied to the potential modeling electrostatic interactions
ref	string	DOI of the reference in which the potential parameters were published
Seventh table: special		
tag1	tag	tag of interaction site (cf. Table 1)
dist	integer	distance between the two sites involved in the special potential given in number of bonds between them
tag2	tag	tag of second interaction site (cf. Table 1)
ID7	integer or “none”	potential function for special potentials encoded in ID_7_
p1	real number	parameter of the special potential function
p2	real number	parameter of the special potential function
…	…	…
ref	string	DOI of the reference in which the potential parameters were published

To avoid redundant or duplicate entries within a section and to keep the tables compact, a short-hand notation is introduced. Thereby, an ‘X’ indicates either a part of a tag or a bond order. The ‘X’ syntax serves as a placeholder for an arbitrary entry. For example, the bond identifier (tag 1, order, tag 2) = (A-C-X-X, 1, A-C-X-X) specifies all types of bonds in alkanes. Hence, they would all be modeled by the same mathematical function and parameters.

### Application of data format

The TUK-FFDat format proposed in this work is applied to three transferable force fields of different type. The three transferable force fields are:

- the TraPPE-UA force field^32–43^ (semi-flexible, united-atom),
- the OPLS-AA force field^44–48^ (flexible, all-atom), and
- the Potoff force field^49–52^ (semi-flexible, united-atom).

The TraPPE-UA and the Potoff transferable force field have been developed within the chemical engineering community. They are widely used for predicting thermodynamic properties – in particular of hydrocarbons^32,33,49,50^. The OPLS-AA transferable force field has been developed within the molecular biology community and is accordingly mostly used for modeling bio systems, e.g. predicting structural protein properties^13^.

The TUK-FFDat implementations of all three transferable force fields (TraPPE-UA, OPLS-AA, and Potoff) are available on Zenodo^98^. In the main body of this work, a representative part of the TraPPE-UA transferable force field is depicted and discussed as examples (cf. Tables 12–16). This selection represents the alkane and alcohol part of the TraPPE-UA transferable force field. In the main body of the manuscript (Tables 12–16), the manuscript references are used instead of the DOIs (see online repository^98^).

**Table 12:** First table (intermolecular) of the data format, cf. Tables 10, 11, for the TraPPE-UA for field for alkanes and alcohols.

tag	ID1	p1	p2	p3	ref
A-C-0-0	1	0	148	3.73	^32^
A-C-1-1	1	0	98	3.75	^32^
A-C-2-1	1	0	46	3.95	^32^
A-C-3-1	1	0	10	4.68	^33^
A-C-4-1	1	0	0.5	6.4	^33^
Ak-O-2-1	1	−0.7	93	3.02	^35^
Ak-H-1-1	1	0.435	0	0	^35^
Ak-C-1-1	1	0.265	98	3.75	^35^
Ak-C-2-1	1	0.265	46	3.95	^35^
Ak-C-3-1	1	0.265	10	4.33	^35^
Ak-C-4-1	1	0.265	0.5	5.8	^35^

**Table 13:** Second table (bonds) of the data format, cf. Tables 10, 11, for the TraPPE-UA force field for alkanes and alcohols.

tag1	order	tag2	ID2	p1	ref
X-C-X-1	1	X-C-X-1	none	1.54	^32^
Ak-C-X-X	1	Ak-O-2-1	none	1.43	^35^
Ak-H-1-1	1	Ak-O-2-1	none	0.945	^35^

**Table 14:** Third table (angles) of the data format, cf. Tables 10, 11, for the TraPPE-UA force field for alkanes and alcohols.

tag1	order1	tag2	order2	tag3	ID3	p1	p2	ref
X-C-X-X	1	X-C-2-1	1	X-C-X-X	1	62500	114	^32^
X-C-X-X	1	X-C-3-1	1	X-C-X-X	1	62500	112	^33^
X-C-X-X	1	X-C-4-1	1	X-C-X-X	1	62500	109.47	^33^
X-C-X-X	1	Ak-C-X-1	1	Ak-O-2-1	1	50400	109.47	^35^
Ak-C-X-1	1	Ak-O-2-1	1	Ak-H-1-1	1	55400	108.5	^35^

**Table 15:** Fourth table (torsion) of the data format, cf. Tables 10, 11, for the TraPPE-UA force field for alkanes and alcohols.

tag1	order1	tag2	order2	tag3	order3	tag4	ID4	p1	p2	p3	p4	ref
X-C-X-X	1	X-C-2-1	1	X-C-2-1	1	X-C-X-X	1	0	355.03	−68.19	791.32	^32^
X-C-X-X	1	X-C-2-1	1	X-C-3-1	1	X-C-X-X	1	−251.06	428.73	−111.85	441.27	^33^
X-C-X-X	1	X-C-2-1	1	X-C-4-1	1	X-C-X-X	1	0	0	0	461.29	^33^
X-C-X-X	1	X-C-3-1	1	X-C-3-1	1	X-C-X-X	1	−251.06	428.73	−111.85	441.27	^33^
X-C-X-X	1	X-C-2-1	1	X-C-3-2	1	X-C-X-X	1	0	0	0	461.29	^33^
X-C-X-X	1	Ak-C-2-1	1	Ak-O-2-1	1	Ak-H-1-1	1	0	209.82	−29.17	187.93	^35^
X-C-X-X	1	Ak-C-3-1	1	Ak-O-2-1	1	Ak-H-1-1	1	215.96	197.33	31.46	−173.92	^35^
X-C-X-X	1	Ak-C-4-1	1	Ak-O-2-1	1	Ak-H-1-1	1	0	0	0	163.56	^35^
X-C-X-X	1	X-C-2-X	1	X-C-2-1	1	X-O-2-1	1	0	176.62	−53.34	769.93	^35^
X-C-X-X	1	X-C-X-1	1	X-O-2-1	1	X-C-X-1	1	0	725.35	−163.75	558.2	^36^
X-O-2-1	1	X-C-2-1	1	X-C-2-1	1	X-O-2-1	1	503.24	0	−251.62	1006.47	^36^

**Table 16:** Seventh table (special) of the data format, cf. Tables 10, 11, for the TraPPE-UA force field for alkanes and alcohols.

tag1	dist	tag2	ID7	p1	ref
Ak-O-X-X	4	X-H-1-1	1	75000000	^36^
Ak-O-X-X	5	X-H-1-1	1	75000000	^36^

The TraPPE-UA transferable force field is a semi-flexible united-atom force field. In the TraPPE-UA force field, all bonds between interaction sites are constrained to be rigid. This translates in the data format as none entries in the second data format table, cf. Table 13. The TraPPE-UA transferable force field does not contain improper torsion potentials. Accordingly, the fifth table of the data format remains empty (not shown). Despite the fact that the TraPPE-UA is a united-atom force field, hydrogen atoms are explicitly modeled in some chemical structures, e.g. specific polar functional groups. Details are given in the Supplementary Material.

## Discussion

A generalized data scheme for transferable force fields was presented that can be applied to various types of force fields such as rigid and flexible as well as all-atom and united-atom force fields. The data scheme is implemented into an SQL-based file format. Thereby, the data scheme is fully machine readable and provides uniquely defined data structures. It is called TUK-FFDat. The TUK-FFDat data scheme and data format is specifically designed for transferable force fields (opposite to component-specific force fields), i.e. it provides data structures for generalized chemical construction plans that define model building blocks for substance classes. Three applications of the data scheme and data format are given (the TraPPE-UA, OPLS-AA, and Potoff transferable force fields). These three examples show important differences, which demonstrates the general applicability of the data scheme. The data scheme and data format proposed in this work can be favorably used for increasing the force field interoperability in the molecular simulations community. The data scheme and data format can be used for sharing transferable force field data between different actors, e.g. database developers, force field developers, and simulators.

The data scheme and data format presented here can readily be extended in different directions. New interaction potentials can easily be added in the corresponding potential lists (cf. Tables 3–8) by adding a new ID_*i*_-value. Also, new chemical groups can be added in the corresponding functional groups list, cf. Table 2. Also, in the case that the topology of the transferable force field is to be extended, new sections can be added to the data scheme. Also, the ongoing development of a given transferable force field can favorably be carried out based on the data scheme by adding entries in the different section tables. If new interaction site types are added to a transferable force field, the new entries specifying the different potential interactions can be readily appended in the lists of the seven sections. For future work, the data scheme proposed in this work can be extended to coarse grain, reactive, and machine learned force fields.

## Methods

### Conversion tools

The SQL-based data format presented here can be favorably used for process automation. For human interaction and creating the tables, the classical .xls spreadsheet format can, however, be more convenient. An auxiliary tool is provided in the online repository^98^ for converting the data scheme from the .xls format to the SQL-based format and vice versa. Therefore, two Python scripts are provided in the online repository^98^. For testing, example .xls and SQL transferable force field files are also provided. The script named xlsx2SQL.py reads an .xls spreadsheet file in which a transferable force field is defined and creates an SQL database containing the corresponding transferable force field. The second script reads a transferable force field from an SQL database and creates the corresponding .xls spreadsheet files. The handling of these scripts is described in detail in the Supplementary Material. The .xls spread files are intended for constructing the actual SQL-based data format files of a given transferable force field.

## Supplementary information

Supplementary Information

## Data Availability

The implemented force field files are publicly available in an online repository^98^.

## Code availability

The code used for converting the data format files and building the SQL-based format are publicly available in an online repository^98^.
