# Supplementary material for: Data scheme and data format for transferable force fields for molecular simulation
Source: Sci Data. 2023 Jul 27;10:495. doi: 10.1038/s41597-023-02369-8 (PMC10374650; doi:10.1038/s41597-023-02369-8)
Supplement: Supplementary file 1 — Supplementary Information [file 41597_2023_2369_MOESM1_ESM.pdf]

SUPPLEMENTARY MATERIAL

Data scheme and data format for transferable force fields for molecular simulation

Gajanan Kanagalingam, Sebastian Schmitt, Florian Fleckenstein and Simon Stephan
Laboratory of Engineering Thermodynamics (LTD), RPTU Kaiserslautern, Kaiserslautern, Germany
Simon.Stephan@rptu.de

2023-07-06

Contents

1 Further Details on the data scheme and data format **2**

1.1 Tags of Interaction Sites Modeling Hydrogen Atoms in All-Atom Transferable Force Fields . . . 2

1.2 Tags of Interaction Sites Modeling Atoms in Cyclic Molecular Structures . . . 3

1.3 Non-Transferable Models of Molecules in the Data Scheme . . . 3

1.4 Details on the Implementation of Rigid, Flexible, and Semi-Flexible Force Fields . . . 4

1.5 Specifications for Special Cis/Trans Isomerism-Dependent Torsion Potentials . . . 4

2 The Data Scheme and Data Format Applied to the TraPPE-UA, OPLS-AA, and Potoff Force Field **4**

3 Building a TUK-FFDat Force Field File **4**

4 The Handling of the Conversion Tools **5**

1 Further Details on the data scheme and data format

1.1 Tags of Interaction Sites Modeling Hydrogen Atoms in All-Atom Transferable Force Fields

The tags for interaction sites modeling a hydrogen atom in all-atom force fields have a special format regarding the last two parts of the tag format. Since hydrogen atoms can only form a single first-order bond, hydrogen atoms would have tags of the form "part1-part2-1-1". To increase the quality of information in the tag, the last two parts of the tag are modified for hydrogen atoms: The third part of the tag thereby describes, which atom is modeled by the interaction site to which the hydrogen atom under consideration is bound. The last part of the tag is a tuple divided by a backslash "\". The first part of the tuple specifies the number of bonds of the interaction site to which the hydrogen atom under consideration is bound. In case of non-cyclic molecular structures, the second part of the tuple describes the highest bond order of the interaction site to which the hydrogen atom under consideration is bound. Therein, the number of bonds neglects bonds to hydrogen atoms. Fig. S1 shows an all-atom model of a n-pentane molecule, in which each interaction site has been assigned its corresponding tag. For cyclic molecular structures see the following section.

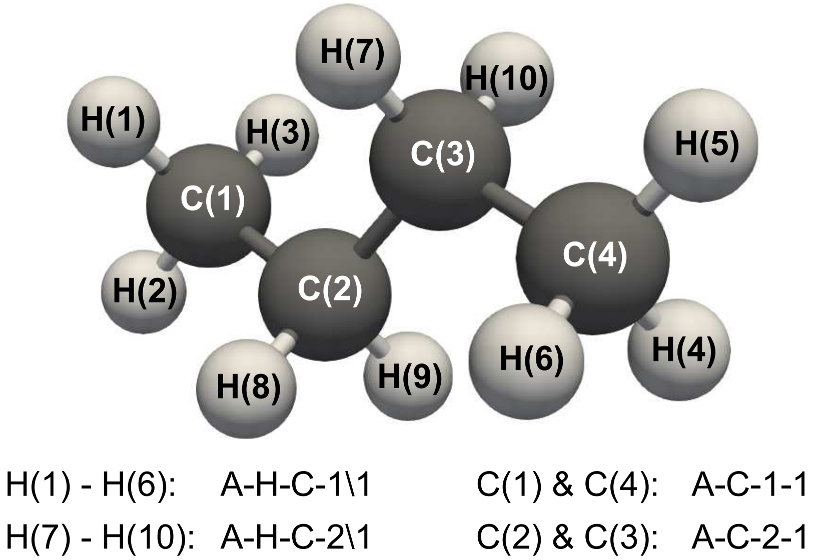

Figure S1. All-atom model of the n-pentane molecule with tags assigned to the interaction sites.

1.2 Tags of Interaction Sites Modeling Atoms in Cyclic Molecular Structures

The tags for interaction sites modeling an atom or a group of atoms within a cyclic molecular structure have a special format regarding the last two parts of the tag format. The second part of the tag is preceded by a "C" to indicate that the interaction site under consideration is located in a cyclic molecular structure. The last part of the tag, on the other hand, consists of several flags separated by a hyphen "/". The first flag describes the functional group within the ring. Table S1 gives examples of flags used in the data format.

Further flags are an alphabetical listing of all second parts of the tags of the interaction sites bond to the interaction site under consideration. In the case that the part of the considered interaction site is "CC", another flag is appended. This appended flag describes the minimum distance of the considered interaction site to another site within the ring whose second part of the tag is not "CC". In case of a pure "CC" ring, the flag has the value "0". Fig. S2 shows a model of an united-atom cyclic molecular structure and the tags associated with each interaction site. For all-atom modeled hydrogen atoms that are bound to a cyclic molecular structure, only the last tag is altered compared to the description in the previous section. The last tag follows the same rules described in this section.

1.3 Non-Transferable Models of Molecules in the Data Scheme

Some transferable force fields also include models of molecules that are intrinsically non-transferable and parameters are fitted for each component-specific force field model. Hence, for specific molecules, exceptions from the transferable model framework are defined and component-specific models are used. For example, water molecules often possess parameters which are exclusively used for this particular molecular model and cannot be applied to any other molecule. The data scheme presented in this work is capable of incorporating these component-specific force field models by assigning them a new functional group. Therefore, the first tag is an abbreviation representing the functional group. The other tags follow the principles of the data scheme outlined in the main body of the paper. Analogously, interaction sites modeling an atom or

Table S1. Flags representing a functional group within a cyclic molecular structure. * Only for cycloalkanes with a ring size of five or six. ** Only for cycloalkanes with a ring size of six.

abbreviation	functional group
CA	cycloalkane
E*	ether
diE(1,3)*	1,3 di-ether
diE(1,4)*	1,4 di-ether
triE**	tri-ether
Aro	benzene

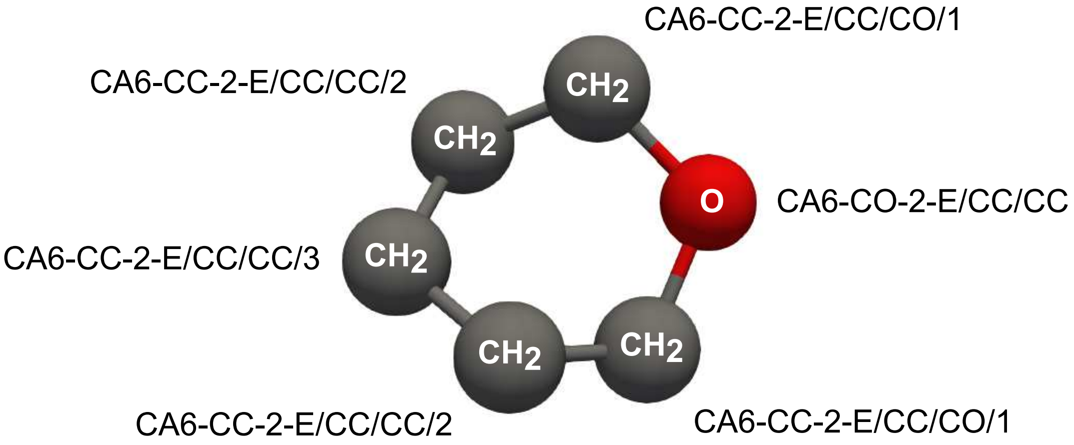

Figure S2. Model of a cyclic molecular structure and the tags associated with the interaction site.

group of atoms within a cyclic molecular structure in a non-transferable model can be assigned a new flag representing the functional group.

1.4 Details on the Implementation of Rigid, Flexible, and Semi-Flexible Force Fields

For rigid force fields, all bond lengths, bond angles or torsion angles are constrained. Semi-flexible force fields allow that individual bond lengths, bond angles or torsion angles are constrained. The TraPPE-UA force field for example uses rigid bond lengths for all bonds, whereas bond angles and torsion angles are not constrained – in most cases. Certain molecular structures (e.g. benzene) are also constrained with respect to their bond angles and torsion angles. Constrained bond lengths or bond angles are given the $\mathrm{ID}_i = 0$ and the parameter p_0 takes either the constrained bond length or the constrained bond angle. All other parameters take the value '-'. In the case of a constrained torsion angle, both the potential and all parameters take the value '-'.

1.5 Specifications for Special Cis/Trans Isomerism-Dependent Torsion Potentials

To distinguish between cis and trans configurations, further metadata is required. Therefore, for isomerism-dependent torsion potentials, the potentials for both configurations are given by specifying two potential functions, parameter sets and meta data separated by a slash (see electronic Supplementary Material). The first data refers to the cis configuration and the second to the trans configuration.

2 The Data Scheme and Data Format Applied to the TraPPE-UA, OPLS-AA, and Potoff Force Field

The TraPPE-UA, OPLS-AA, and Potoff transferable force fields were implemented in the TUK-FFDat data format. The corresponding files are provided in the electronic Supplementary Material in the three files "`TUK-FFDat_TraPPE-UA.xlsx`", "`TUK-FFDat_OPLS-AA.xlsx`", and "`TUK-FFDat_Potoff.xlsx`".

It should be noted that the extension of the TraPPE-UA force field from Zhang et al. (2005) [full reference given in the main body of this work] was used, since perflouranes have a backbone consisting of "$\mathrm{CF_x}$" interaction sites instead of "$\mathrm{CH_x}$" interaction sites. In the special case of perflouranes, each interaction site "$\mathrm{CF_x}$" is assigned the first and second part of the interaction site tag "A-PF" instead of the regular alkane tag of "A-C".

3 Building a TUK-FFDat Force Field File

For force field developers, we provide a blank .xlsx TUK-FFDat template (`template.xslx`) that can be used to create new transferable force field files. The template consists of seven tabs (`intermolecular`, `bond`, `angle`, `torsion`, `improper`, `1n_potential`, `special`) – analogue to the interaction types (cf. Tables 2–8 in the main body). The headers of the tables are colored in dark green in the template. The names of the headers may not be changed as they are part of the data format. The body of the table is colored light green. This body needs to be filled with the actual force field data as outlined in Tables 2–8 in the main body. Examples how the force field data needs to be filled are given via the TraPPE, OPLS-AA, and Potoff force field (see above). For each of the seven tabs, the columns defined by the data format are present. Since the number of parameters is variable for each interaction, additional columns can be added (see Table 2-8, 10 and 11 in

the main body for details). In the template, placeholders (orange colored) for additional columns are added for clarity. The parameters have to be numbered in ascending order (p1, p2, p3, ...) and its names have to be inserted in the respective header cells. Hence, the number of columns varies according to the number of parameters used in a given force field. Moreover, the number of parameters and columns is indirectly coded in the ID variable of each tab. Any number of rows can be used within the limits of the .xlsx file format. The .xlsx file can then be converted to an SQL database with the tools described in the next section.

4 The Handling of the Conversion Tools

The python scripts "xlsx2SQL.py" and "SQL2xlsx.py" are designed to run under python 3. The requirements for running the scripts given in the electronic Supplementary Material is a computer on which python 3 and the package manager "pip" are installed. Further python modules needed to run the scripts "xlsx2SQL.py" and "SQL2xlsx.py" that are specified in the file "requirements.txt" and can be installed with the help of the package manager "pip". Using the command line, the directory in which the file "requirements.txt" was downloaded needs to be navigated to. The following command is then to be entered into the command line:

```
$ pip install -r requirements.txt
```

Both python scripts "xlsx2SQL.py" and "SQL2xlsx.py" require two arguments each. The script "xlsx2SQL.py" reads in an .xlsx spread sheet file containing a transferable force field in the TUK-FFDat data format and outputs an SQL script that sets up a corresponding SQL database containing the transferable force field. The first argument to this script is the name of the .xlsx spread sheet file including the file extension ".xlsx". The second argument is the name of the SQL script to be generated – also including the corresponding file extension ".sql". The .xlsx spread sheet file should have the same structure as the files .xlsx given in the Supplementary Materials, which means that the names and headers of the individual tables must be identical.

The python script "SQL2xlsx.py" is the counterpart to the "xlsx2SQL.py" script. It reads in an SQL script that sets up a database and then outputs an .xlsx spread sheet file. The first argument to this script is the name of the SQL script including the file extension ".sql". The second argument is the name of the .xlsx spread sheet file to be generated – also including the corresponding file extension ".xlsx".

Thus, the two scripts "xlsx2SQL.py" and "SQL2xlsx.py" can be executed with the following commands via the command line:

```
$ python3 xlsx2SQL.py input.xml output.sql
```

or accordingly:

```
$ python3 SQL2xlsx.py input.sql output.xml
```
